# Kratom: A Narrative Review of the Possible Clinical Uses and Dangers of This Opioid-Like Plant

**DOI:** 10.7759/cureus.73058

**Published:** 2024-11-05

**Authors:** Amber N Edinoff, Sarah E Kaufman, Taylor C Mahoney, William C Upshaw, Jay Gong, Elyse M Cornett, Kevin S Murnane, Adam M Kaye, Giustino Varrassi, Sahar Shekoohi, Alan D Kaye

**Affiliations:** 1 Psychiatry, Harvard Medical School, Boston, USA; 2 Alcohol, Drugs, and Addiction, McLean Hospital, Belmont, USA; 3 Psychiatry and Behavioral Medicine, Louisiana State University Health Sciences Center Shreveport, Shreveport, USA; 4 School of Medicine, Louisiana State University Health Sciences Center New Orleans, New Orleans, USA; 5 School of Medicine, Louisiana State University Health Sciences Center Shreveport, Shreveport, USA; 6 School of Medicine, Tulane University, New Orleans, USA; 7 Anesthesiology, Louisiana State University Health Sciences Center Shreveport, Shreveport, USA; 8 Pharmacology, Toxicology, and Neurosciences, Louisiana State University Health Sciences Center Shreveport, Shreveport, USA; 9 Louisiana Addiction Research Center, Louisiana State University Health Sciences Center Shreveport, Shreveport, USA; 10 Pharmacy Practice, Thomas J. Long School of Pharmacy and Health Sciences, University of the Pacific, Stockton, USA; 11 Pain Medicine, Paolo Procacci Foundation, Rome, ITA; 12 Anesthesiology and Pharmacology, Toxicology, and Neurosciences, Louisiana State University Health Sciences Center Shreveport, Shreveport, USA

**Keywords:** internet medications, kratom, opioid-like, supplements, withdrawal

## Abstract

The term "kratom" refers to a plant species formally known as *Mitragyna speciosa. *Kratom is composed of over 40 alkaloids, a type of organic compound that contains nitrogen. These compounds work primarily via binding to opioid receptors expressed on neurons, where they stimulate signal transduction mechanisms involving the activation of G proteins. Kratom has been shown to cause both a stimulant-like effect and a sedative effect in humans. These studies have shown that use is highest among European-American, middle-class men living in suburban areas.

Additionally, individuals who have a history of opioid misuse are also more likely to take kratom. Kratom is used by many different people in the US for numerous different reasons. Some of the most often cited reasons include treating chronic pain conditions, depression, and anxiety. Individuals who used kratom for these reasons typically consumed kratom daily at a dose of 1-3 grams, with the kratom extracted into a powder to be consumed in a capsule. Additionally, there have been reports of kratom being used to treat opioid withdrawal symptoms, as kratom can bind to some of the same receptors as opioids. This manuscript specifically describes trends regarding the use of kratom in the US, pharmacokinetic and pharmacodynamic properties of kratom, potential therapeutic uses of kratom, adverse events caused by kratom, and case studies in the literature regarding patients using kratom.

## Introduction and background

Kratom is a plant-derived from a species of plant native to Southeast Asia called *Mitragyna speciosa *[1]. It is generally prepared through boiling leaves derived from *Mitragyna speciosa* to produce kratom tea. Additionally, kratom can be crushed in order to produce a powder that is then able to be ingested [2]. Kratom comprises numerous alkaloids, which are organic compounds that contain nitrogen. Two of these alkaloids are mitragynine and 7-hydroxy mitragynine. Both are believed to be crucial in kratom's physiological effect on humans [3]. Though the mechanism of action of these compounds is still not fully understood, it is believed they work primarily via acting as agonists at certain subtypes of opioid receptors expressed on neurons, with mitragynine acting at the μ and δ receptors while 7-hydroxy mitragynine acts at the μ and κ receptors. These receptor-ligand interactions may then stimulate signal transduction mechanisms involving the activation of G proteins. In addition, these metabolites are also believed to act on various monoaminergic and opioid receptors [4,5].

Kratom has been shown to cause both a stimulant-like effect and a sedative effect in humans. The physiological effects vary based on the dose that is taken as well as the particular strain of kratom that is consumed [3]. For example, the red vein strain of *Mitragyna speciosa* tends to induce a sedated state when ingested, whereas the green vein strain tends to cause a stimulating effect [6]. The use of kratom has historically been by people living in Southeast Asia [7]. However, in recent years, the use of kratom has increased significantly among people living in the United States [8]. In the United States, kratom can now be bought online or at certain drug shops [9]. As kratom has only recently gained popularity in the United States, much is still unknown regarding the potential adverse effects of the drug. However, some negative side effects that have been documented include nausea, vomiting, hypertension, tachycardia, and elevated levels of creatine phosphokinase [10].

Additionally, there have been several instances where kratom intake caused severe liver damage that was eliminated once patients stopped consuming kratom [11]. Further compounding issues with kratom are evidence showing that individuals taking kratom develop a physiological dependence, especially when taking other drugs of abuse, which can result in withdrawal symptoms. These withdrawal symptoms include extreme pain, muscle spasms, and difficulty sleeping [12,13]. This drug, even though outlawed in some states, is still easily obtainable, and clinicians should be aware of the use and potential issues associated with the use of this drug. This article will specifically discuss trends regarding the use of kratom in the US, pharmacokinetic and pharmacodynamic properties of kratom, potential therapeutic uses of kratom, adverse events caused by kratom, and case studies in the literature regarding patients using kratom.

## Review

Kratom in the United States

Kratom has been used for an extended period in Southeast Asian countries such as Malaysia, Thailand, and Cambodia. However, kratom has only recently gained popularity in countries in the Western hemisphere, such as the United States (US) [7]. The use of kratom was first noted in the US beginning in the mid-2000s, but the use of this plant has become significantly more widespread [14,15]. There were many unsuccessful attempts to ban the sale of kratom on the federal level. Instead, kratom remains classified by the Drug Enforcement Administration (DEA) as a drug and chemical of concern [16]. While there is no federal law prohibiting the sale of kratom, its sale is still illegal in six states. However, in states where the sale of kratom is not prohibited, the drug is sold at smoke shops, online stores, and convenience stores, creating a widespread availability of the drug to the public [17].

Studies have detailed what demographic in the US displays the highest rate of kratom consumption. These studies have shown that use is highest among White, middle-class men living in suburban areas. Additionally, individuals who have a history of opioid misuse are also more likely to take kratom [18]. Another risk factor for kratom use that has been found is the use of e-cigarettes. A study found that individuals who reported using e-cigarettes within the past year had 4.8 higher odds of using kratom within that same interval than those who did not [19]. This is important to note as the use of e-cigarettes has skyrocketed in recent years, especially among teenagers and young adults [20].

Kratom is used by many different people in the US for numerous other reasons. Some of the most often cited reasons include to treat chronic pain conditions, depression, and anxiety. Individuals who used kratom for these reasons consumed kratom daily at a typical dose of 1-3 grams, with the kratom converted into a powder so that it could be consumed in a capsule [21]. Kratom may also be used by people due to its stimulant-like effects, causing increased feelings of motivation, higher levels of energy, a stronger desire to work, and an overall increase in productivity [22]. Additionally, there have been reports of kratom being used to treat opioid withdrawal symptoms, as kratom can bind to some of the same receptors as opioids [14,23].

As the popularity of kratom has risen in the US, concerns about potential adverse effects have also increased. Studies have shown numerous unwanted effects of kratom use, such as sinus bradycardia, tachycardia, constipation, diarrhea, headache, and intracerebral hemorrhage [15,24-27]. Additionally, withdrawal symptoms have been noted among people in the US attempting to cease using kratom. These withdrawal symptoms strongly resemble those experienced by patients who stop using opioids after a period of use that has induced physical dependence [12]. Furthermore, fatalities have been thought to be partially due to ingesting an exceedingly large amount of kratom. In most of these cases, however, there were usually other drugs found in the deceased individuals' system, making the exact cause of death difficult to determine [28].

Kratom and other substances used for disease management

There is no acknowledged medical use for kratom in the US [29]. However, kratom is used extensively in other countries, such as many of those found in Southeast Asia. There, kratom is used to treat a wide range of many different conditions. Some of these conditions kratom is used to treat include diarrhea, diabetes, poor blood circulation, and to reduce cravings for various addictive drugs [30,31].

Additionally, there is hope that kratom may be able to be used as an antidepressant and antipsychotic, as certain alkaloids within the drug can alter serotonin and dopamine signaling pathways [32]. Further research supporting using kratom as an antipsychotic was conducted using a mouse model [33]. In this study, kratom was shown to reverse both the positive and negative symptoms of schizophrenia-like behavior. Along with this, research supporting the use of kratom as an antidepressant has come from studies showing a decreased immobility time in the forced swimming test, a test commonly performed to test the efficacy of new antidepressant drugs. It is believed this effect may have been caused by kratom increasing activity in the dorsal raphe nucleus, which is crucial for the production in the brain of the neurotransmitter serotonin [34].

Another proposed use for kratom is to treat opioid withdrawal symptoms, for which this drug is already used in some parts of the world [35]. There is some evidence that kratom may help treat issues related to opioid withdrawal. Animal studies involved the induction of morphine dependence in mice. The administration of morphine was then abruptly discontinued to precipitate symptoms of opioid withdrawal in the mice, which was significantly attenuated by the administration of mitragynine [36,37]. Kratom may be able to serve this function due to its action at the mu-opioid receptor [38]. Many of the more severe side effects of opioids, such as respiratory depression, can be generally avoided with kratom administration [10]. That being said, there is still evidence of issues arising from chronic kratom intake. These include the development of dependence on kratom, tolerance to its effects, and withdrawal symptoms that occur upon discontinuation of the drug after using it for an extended period [39]. Thus, even though kratom has shown evidence of potentially being a helpful drug to help patients cease taking opioids, more research should be done on its long-term effects before it starts being widely prescribed.

Kratom mechanisms of action

Kratom contains over 50 naturally occurring alkaloids, with variations in alkaloid content based on the location and the climate conditions of the areas where the leaves are grown [40]. The primary pain-reducing mechanism of action of kratom is through the effects of the alkaloids 7-OH-mitragynine and mitragynine [41]. The 7-OH-mitragynine and mitragynine (along with its diastereomers speciogynine and speciociliatine) have been shown to act on µ opioid receptors, δ opioid receptors, and κ opioid receptors to produce kratom’s analgesic effects at varying binding affinities [42,43]. The 7-OH-mitragynine was approximately 46 times more potent than mitragynine and 13 times more potent than morphine [44]. Additionally, some studies have found that mitragynine may act on receptors for D2 dopamine receptors, serotonin (5-HT2C and 5-HT7) receptors, and alpha-2 adrenergic receptors [40]. Studies on paynantheine and speciogynine, two indole alkaloids also found in kratom, have shown a high affinity for 5-HT1A and 5-HT2B receptors. Definitive in vivo and in vitro studies have shown that various kratom alkaloids or their metabolites have activity at 5-HT1A receptors, which likely contributes to their anxiolytic effects [45]. Although the effects of kratom alkaloids are mainly attributed to only a few pharmacologically active alkaloids, studies on kratom continue to find novel alkaloids with antinociceptive and psychoactive effects [46-49].

As indole alkaloids, kratom alkaloids exhibit similar but different effects as opioids due to their pharmacologic and structural differences. Both indole alkaloids and opioids bind to opioid receptors to initiate G-protein-coupled receptor (GPCR) signaling. However, some indole alkaloids do not initiate the β-arrestin pathway responsible for many symptoms associated with opioid use, such as respiratory depression, sedation, and tolerance [50-52]. For example, mitraciliatine, another alkaloid derived from kratom, demonstrated β-arrestin recruitment at κ opioid receptors but no β-arrestin recruitment at µ opioid receptors [48]. Combined with the adrenergic effects of other indole alkaloids, kratom can potentially provide multimodal pain control with fewer opioid-like adverse effects [40].

Kratom pharmacokinetics/pharmacodynamics

Kratom leaves are typically crushed, smoked, brewed in tea, or eaten [41]. The half-life of mitragynine is around 3.5 hours. The onset of effects of kratom alkaloids is about 10-20 minutes, with peak effects occurring around two to four hours after ingestion [4]. The clinical effects of kratom can vary by dose, producing stimulant-like effects at lower doses and opioid-like effects at higher doses [53,54].

The kratom alkaloids are primarily metabolized hepatically by cytochrome P450 enzymes through linear pharmacokinetics and have a biphasic elimination pattern [55,56]. Mitragynine is metabolized to the more active form 7-OH-mitragynine through four CYP 450 isoenzymes: 2C19, 3A4, 2D6, and 2C18 [56-58]. The CYP3A4-mediated dehydrogenation process is believed to activate mitragynine, not the trace concentrations of 7-OH-mitragynine found in kratom extracts that lead to its analgesic effects [40].

Kratom use is associated with various toxicities and organ dysfunction through studies on cell lines and animal models [43,59]. Studies have found evidence of cardiotoxic and cytotoxic effects of mitragynine and its diastereomers [47,60]. Indole alkaloids have also been shown to inhibit CYP450 isoforms (including CYP3A4 and CYP2D6), resulting in drug-drug interactions with CYP450-mediated compounds [61,62]. Studies also showed that the kratom alkaloids have activity against glutathione transferases, including UGT1A1 and UGT2B7, suggesting interactions with other UGT substrate drugs [63-65]. These interactions can contribute to various presentations associated with kratom toxicity in the form of the airway, antipsychotic toxicities, and organ injury [41].

Case studies

Cases Examining Addiction Presentation and Other Risks

Though kratom is being touted as an addiction-free solution to the opioid epidemic, various case studies examining the relationship between kratom use and addiction appear to contradict the claims. One such study examines the case of a 37-year-old woman with no history of substance use disorder reporting to an inpatient mental health and addiction service requesting treatment for her "kratom addiction." Her kratom use spanned two years; she initially ingested the kratom as capsules containing crushed, dehydrated kratom leaves. Due to pricing concerns, she transitioned to ingesting concentrated kratom extract purchased online. Six months after switching to the concentrated form, she found she was ingesting a much higher quantity of kratom than initially intended and attempted to "cut back." This attempt at cutting back led to cravings and physical withdrawal signs, including severe abdominal cramps, sweats, vision changes, nausea, vomiting, and diarrhea. She attempted outpatient detoxification using low-dose clonidine twice in the next year and a half with no success. Inpatient treatment was only sought after family members confronted her regarding the significant lengths she would use kratom and hide it from her loved ones. Upon admission to an inpatient addiction treatment facility, the patient's lab testing revealed a negative urine toxicology report for all drugs of abuse, including oxycodone, opioids, and methadone. A urine sample examined by liquid chromatography-mass spectrometry was positive for mitragynine, the active alkaloid in kratom. She was started on the program used for opioid withdrawal; this included symptom-triggered clonidine administration every two hours based on the Clinical Opioid Withdrawal Scale (COWS) score. Hydroxyzine was also administered to assist with withdrawal symptoms. By mid-afternoon of the patient's first day in the inpatient program, she was experiencing severe withdrawal symptoms and required 2 mg of clonidine over thirty-six hours. Extended-release venlafaxine was prescribed to combat depression. Hyperautonomic symptoms improved rapidly under treatment, and by the third day of treatment, the woman was considered stable. She was discharged under the care of her husband and father with enrollment in a dual partial hospital program and a prescription for naltrexone 50 mg to combat cravings [66].

Another case study examines a 38-year-old woman with a history of major depressive disorder and opioid use disorder. The patient was reportedly in her eighth year of remission from heroin use with buprenorphine/naloxone when she was taken to the emergency room following an intentional overdose of quetiapine and duloxetine. Her urine drug screen was positive only for cannabis. While her vital signs, including respiratory and heart rates, remained stable throughout hospitalization, her mood fluctuated between somnolence and irritability, anxiety, and crying. She complained of severe anxiety, nausea, myalgias, and abdominal pain; subsequent labs and imaging were normal, and the physical exam was negative for abdominal tenderness. The patient was transferred to the psychiatric hospital, where she revealed she had taken large quantities of kratom for the last eight months. Her kratom use began when she relocated to a new state and could no longer utilize her usual buprenorphine clinic; she intended kratom to serve as a replacement for buprenorphine. Her kratom dose gradually increased as tolerance and cravings increased; as her use continued, she progressed to ingesting an estimated 35 to 42 grams of kratom powder a day (per the American Kratom Association, a dose of 5 or more grams is considered a high dose). Any attempt to wean the kratom resulted in anxiety, muscle spasms, loss of focus, and shakiness. She became depressed, withdrawn, and isolated from loved ones due to her ongoing kratom use. Following the guidelines established by the Diagnostic and Statistical Manual of Mental Disorders, Fifth Edition (DSM-5), her case was diagnosed with severe kratom use disorder. Following admission into the psychiatric hospital, the patient was restarted on her home medications of quetiapine and duloxetine; buprenorphine was initiated on the fifth day of her hospital stay with the agreement that she would resume treatment at a buprenorphine clinic upon release. Symptoms showed slight improvement the two few days in the hospital but eventually showed gradual improvements in mood, anxiety, and body pains. Though kratom withdrawal is thought to resolve within one to three days typically, she remained in the hospital for eight days before experiencing full resolution of her insomnia, anxiety, and abdominal pain. This prolonged treatment time is possibly due to the high dosage being ingested daily. It is unclear the role kratom played in her suicide attempt, as no association has been found between depression and kratom use [67].

In a case series analyzing kratom use in a veteran demographic, four patients with a history of kratom use disorder were studied in conjunction with buprenorphine/naloxone treatment. Prior to treatment, they experienced a wide range of withdrawal symptoms. Patient 1 sought treatment due to the frequent monthly episodes of vomiting that would last up to five days at a time because of his kratom use; once in a treatment program, he described many withdrawal symptoms commonly seen with opioid withdrawal: rhinorrhea, diarrhea, anxiety, restless legs, arm, and leg spasms. Patient 2 reported only rhinorrhea and irritability during withdrawal; Patient 3 experienced anxiety, a foul temperament, and restless legs [68].

Several case studies caution against possible neuropsychiatric risks with kratom use, especially when large doses of kratom are taken for an extended period of time. One such case examines the case of a 27-year-old male with a history of generalized anxiety disorder, attention-deficit/hyperactivity disorder (ADHD), benzodiazepine use disorder, and opioid use disorder. The man reported drinking three to four 8-ml bottles of kratom daily for the last year and a half. He denied any benzodiazepine or opioid use within the last few years; he had no reported increased risk for epilepsy. Though his urine drug screen was positive for benzodiazepines, it is believed this was a false-positive due to the patient's use of diphenhydramine as a sleep aid; the association between diphenhydramine and a false-positive opioid test has been established. His brother brought him to the hospital after walking outside and witnessing him amid a tonic-clonic seizure. Metabolic testing, spinal and brain imaging all returned normal. However, he denied withdrawal symptoms while in the hospital and admitted to experiencing abnormally heightened anxiety, racing thoughts, and insomnia. His care team concluded that he experienced the seizure due to his kratom use, and he was released with anxiolytics after counseling regarding cessation of kratom use [69]. Another case looks at a similar instance of kratom-associated seizures in a man without any previous epilepsy risk factors. A 24-year-old man endures a generalized tonic-clonic seizure following kratom use; all laboratory tests and neuroimaging were normal. He had two more seizures in the following two months after kratom use; he desisted from his use of kratom and has reported no further seizures [70].

Cases Regarding Treatment of Kratom Addiction

Similar to other opioid use disorders, treatment for kratom addiction may involve opioid substitution therapy with methadone or buprenorphine or administration of naltrexone. However, buprenorphine is commonly turned to as a first-line treatment. One case series examines the efficiency of long-term buprenorphine as a treatment for kratom use since it is similar to opioid use disorder. Twenty-eight patients with self-reported kratom use varying lengths (ranging from one month to 25 years) were initiated on buprenorphine treatment. Though the patients required varying stabilizing doses of buprenorphine, there did not appear to be any correlation between the stabilizing dose and the individual's past daily kratom dosage. Of the twenty-eight patients, fifteen reported significant relief in kratom withdrawal symptoms and cravings by the second appointment; the other thirteen participants still had mild to moderate withdrawal that eventually resolved with either an increased buprenorphine dose or additional visits for treatment administration. These patients remained in treatment for 11 months with an average stabilizing dose of buprenorphine/naloxone of 16 mg and a tapered final dose of 14 mg. By the 12th week of treatment, 82% of patients tested negative for mitragynine. Though nine participants admitted to restarting kratom use during treatment, all self-reported using a lower dose than previously used prior to treatment. The authors of the case series concluded that buprenorphine is a promising tool to combat kratom addiction, but further studies with greater power must be done [71].

One combined systemic review and case series also looked at the effectiveness of buprenorphine as a treatment for kratom withdrawal and dependence. Through the analysis of eight cases of patients with kratom use disorder being treated with buprenorphine, this review concluded that not only was buprenorphine an effective treatment for kratom use withdrawal, but there is a strong positive correlation between the dose of daily kratom ingested and the dose of buprenorphine necessary for treatment. This review proposes that for those who use less than 20 mg/day of kratom, an initial dosage between 4 mg and 8 mg buprenorphine/day should be started. For patients using more than 40 mg/day of kratom, an initial dose between 12 mg and 16 mg buprenorphine/day is recommended [72].

In the previously mentioned case series looking at kratom use in a veteran population, there appeared to be a positive trend in kratom withdrawal treatment between increasing dose needs for buprenorphine treatment and polysubstance use. Patient 2, in this case series, required a daily buprenorphine dose of 24 mg, significantly higher than the other cases examined in the series. This need for an increased dose is theorized to be due to his concurrent use of kratom, benzodiazepines, tramadol, and zolpidem. With this dose of buprenorphine, he could wean off all substances being used and was eventually lowered to a stabilized dose of 16 mg buprenorphine/day. Though typically thought that buprenorphine is best initiated twelve to twenty-four hours after the last full dose of opioid agonist to avoid withdrawal induction, the cases examined in this case series indicate that administering buprenorphine as soon as eight hours after the last kratom use can safely occur. The one witnessed induction event occurred when buprenorphine was administered sooner than eight hours post-kratom use. However, the inducted withdrawal symptoms were not exacerbated, as demonstrated by a lower-than-predicted COWS score [68].

Though buprenorphine is the current mainstay treatment of kratom use disorder, one case study reports the success of treatment with a tricyclic antidepressant. Due to the potential toxicity of tricyclic antidepressants, however, it is important that an appropriate dose that is individualized to that specific patient is used. The case describes a 44-year-old man presenting for treatment of kratom withdrawal and heightened anxiety. At the time of the initial interview, he was described as anxious, profusely sweaty, agitated, and emotionally labile. He reported treatment for his anxiety for the last few months with benzodiazepines and selective serotonin reuptake inhibitors (first paroxetine, then sertraline) with no success. He has a history of polysubstance use, with current inappropriate use of benzodiazepines, alcohol, and kratom. Over the last ten months, his kratom use reached a disconcerting level, and, in the two months before seeking treatment, he tried with no avail to cut back on his kratom use. The patient was started on pregabalin 25 mg with a gradual taper off sertraline to be replaced with 150 mg/day bupropion and 300 mg controlled-release trazodone.

Two weeks into this treatment regime, symptoms had not improved, and the patient continued to report restlessness, anxiety, agitation, dysphoria, and insomnia; he also reported continued craving and abuse of benzodiazepines and alcohol. Doses of both pregabalin and bupropion were increased. At his third visit, four weeks into treatment, symptoms still had no improvement. A dose of 50 mg/day of tramadol was added to his treatment regime. Though this reduced his cravings and restlessness, his anxiety remained "paralyzing," and dysphoria persisted. Tramadol dose was increased to 100 mg twice a day, and clomipramine was gradually introduced until a target dose of 75 mg/day was reached. Bupropion was gradually discontinued. After one month on the new treatment regime, the patient reported significantly declining cravings and anxiety levels; tramadol was discontinued. In the following months, all clinical symptoms, cravings, and anxiety were reduced to a level allowing for discontinuation of all medications apart from the 75 mg/day clomipramine. Nine months following the case study, the patient reported the resolution of all cravings and anxiety with a return to his "normal life" [73].

Not many cases are reported on kratom use disorder treatment in young adults. One such case describes a 20-year-old male with a history of ADHD attempting at-home treatment of kratom use disorder with buprenorphine treatment. The patient presented for treatment due to emotional bluntness following kratom use and an inability to take his ADHD medication due to intolerable cardiac symptoms (tachycardia, heart palpitations). Initial dosing at 4 mg of buprenorphine resulted in the young man calling emergency services with reports of dizziness and dysphagia with denial of shortness of breath, chest pain, or palpitations. The dose of buprenorphine was lowered to 2 mg daily for four days before the patient began to experience withdrawal symptoms. Buprenorphine was increased back to 4 mg daily. He remained at this dose for one month before attempting unsuccessfully to decrease his dose to 3 mg; he continued at a dose of 4 mg for three more months with a single incident of kratom use relapse. In another attempt to decrease the dose, a treatment plan was established in which the patient alternated between 4 mg and 2 mg buprenorphine daily every other day. With this plan of action, the patient could resume his ADHD medication. At the time of the publication of the case study, the patient was continuing at this dosage with the reported resolution of all cravings and withdrawal symptoms; his care team hopes to be able to taper him off entirely by year's end [74]. It will be interesting to study how this drug interacts with the sigma receptors, especially in view of their neuroprotective action [75].

## Conclusions

Kratom use in the US is not approved for any reason. However, there are medical uses in other countries. The pharmacologic profile and range of effects of kratom require more oversight, as there is limited knowledge about the risks associated with it and insufficient information to counsel patients on its benefits. Kratom offers potentially desirable benefits for pain control compared to the current opioids, particularly the withdrawal symptoms and its side effect profile. However, the various psychoactive effects of indole alkaloids may result in significant drug-drug interactions in patients on other medications. These interactions are particularly concerning given that kratom is used predominately as a self-treatment nutraceutical that patients use for various medical problems. This creates a need for physicians to be familiar with kratom and its potential interactions, as well as a growing need for medical providers to recognize the signs and symptoms of kratom use, addiction, intoxication, or withdrawal. Given the potential adverse effects, there may be a need for more control over kratom use to limit accessibility.

While some excellent studies have already been published, there remains a strong need for more high-quality research on kratom and the effects of its components to better guide its use in the current analgesic landscape in the US. It is essential to elucidate the effects and better understand kratom's potential activities at various doses, as well as to determine the effects of the various individual alkaloids within the kratom plant. There is also a need to provide patients with a better understanding of potential risks when using the substance, particularly in individuals who choose to self-medicate with kratom. Further extensive research on kratom is needed to determine if it has a role in the medical management of pain and potentially more in the US.
